# Minimising Immunohistochemical False Negative ER Classification Using a Complementary 23 Gene Expression Signature of ER Status

**DOI:** 10.1371/journal.pone.0015031

**Published:** 2010-12-01

**Authors:** Qiyuan Li, Aron C. Eklund, Nicolai Juul, Benjamin Haibe-Kains, Christopher T. Workman, Andrea L. Richardson, Zoltan Szallasi, Charles Swanton

**Affiliations:** 1 Center for Biological Sequence Analysis, Technical University of Denmark, Lyngby, Denmark; 2 Computational Biology and Functional Genomics Laboratory, Harvard School of Public Health, Center for Cancer Computational Biology, Dana-Farber Cancer Institute, Boston, Massachusetts, United States of America; 3 Department of Pathology, Brigham and Women's Hospital, Boston, Massachusetts, United States of America; 4 Children's Hospital Informatics Program at the Harvard-MIT Division of Health Sciences and Technology (CHIP@HST), Harvard Medical School, Boston, Massachusetts, United States of America; 5 Translational Cancer Therapeutics Laboratory, Cancer Research UK London Research Institute, London, United Kingdom; 6 Breast and Drug Development Units, Royal Marsden Hospital, Sutton, United Kingdom; Health Canada, Canada

## Abstract

**Background:**

Expression of the oestrogen receptor (ER) in breast cancer predicts benefit from endocrine therapy. Minimising the frequency of false negative ER status classification is essential to identify all patients with ER positive breast cancers who should be offered endocrine therapies in order to improve clinical outcome. In routine oncological practice ER status is determined by semi-quantitative methods such as immunohistochemistry (IHC) or other immunoassays in which the ER expression level is compared to an empirical threshold[1], [2]. The clinical relevance of gene expression-based ER subtypes as compared to IHC-based determination has not been systematically evaluated. Here we attempt to reduce the frequency of false negative ER status classification using two gene expression approaches and compare these methods to IHC based ER status in terms of predictive and prognostic concordance with clinical outcome.

**Methodology/Principal Findings:**

Firstly, ER status was discriminated by fitting the bimodal expression of ESR1 to a mixed Gaussian model. The discriminative power of ESR1 suggested bimodal expression as an efficient way to stratify breast cancer; therefore we identified a set of genes whose expression was both strongly bimodal, mimicking ESR expression status, and highly expressed in breast epithelial cell lines, to derive a 23-gene ER expression signature-based classifier. We assessed our classifiers in seven published breast cancer cohorts by comparing the gene expression-based ER status to IHC-based ER status as a predictor of clinical outcome in both untreated and tamoxifen treated cohorts. In untreated breast cancer cohorts, the 23 gene signature-based ER status provided significantly improved prognostic power compared to IHC-based ER status (P = 0.006). In tamoxifen-treated cohorts, the 23 gene ER expression signature predicted clinical outcome (HR = 2.20, P = 0.00035). These complementary ER signature-based strategies estimated that between 15.1% and 21.8% patients of IHC-based negative ER status would be classified with ER positive breast cancer.

**Conclusion/Significance:**

Expression-based ER status classification may complement IHC to minimise false negative ER status classification and optimise patient stratification for endocrine therapies.

## Introduction

Breast cancer is classified into clinically relevant subtypes based on the expression of the oestrogen receptor (ER), classifying tumours into ER positive and ER negative cases. These subtypes are characterized by fundamentally different clinical risk for disease-specific survival and response to various therapies[3]. ER positive tumours are generally associated with better prognosis than ER negative tumours and respond well to endocrine therapies affecting oestrogen receptor activity. On the other hand, ER negative tumours are highly proliferative and insensitive to endocrine therapies. Consequently, the correct classification of ER status, with particular attention to minimising the false negative rate, has far reaching clinical implications in prognostication and patient stratification for treatment. In current clinical practice, ER expression levels are measured by semi-quantitative methods such as immunohistochemistry (IHC) or enzymatic immunoassay (EIA). To determine the ER status of a given tumour, an empirical, subjectively chosen threshold has to be set. There are major drawbacks of such a method; firstly, the analytical set-up is difficult to standardize across laboratories. Secondly, aspects of the staining protocols such as the length of antigen retrieval and tissue fixation differ from centre to centre resulting in a significant level of variation in ER status classification[4]; thirdly, the ER status derived from immunostaining approaches remains a subjective judgement[5]; finally, the relationship between the empirical threshold of ER positivity and the true underlying biological function of the receptor, which is likely to determine endocrine therapy sensitivity, is poorly elucidated[4]. These factors together may result in a significant level of discordance of ER status classification with a major impact on treatment choice and clinical outcome in breast cancer.

Clinical studies of breast cancer have suggested that microarray-based gene expression profiling may serve as a robust alternative to immunohistochemistry to determine ER status in breast cancer[5], [6], [7], [8]. Furthermore, the abundance of genes quantified by high throughput profiling has led to the discovery of a complex molecular network regulated by ER[9]. However, determination of the optimal threshold for gene expression-based ER predictors still remains problematic. One approach is to compare microarray based expression measurements of the ER to those of IHC and define the threshold value as the probe level that best separates ER positive from ER negative tumours, determined according to conventional methodologies[5]. Another approach is to select genes highly correlated with ESR1 and define molecular subtypes corresponding to pathological ER status based on the bimodal distribution of the expression levels of a selected gene set[10], [11].

These expression-based methods yield generally consistent classifications in most of the samples tested between selected ESR1 probe levels and the corresponding ER expression measured by IHC. However, the concordance of the two methods varies from one data set to another. Indeed, for a significant proportion of samples, gene expression based classification and IHC based classification differ, and it is currently unclear whether these cases behave clinically more like true ER positive or true ER negative cases[5]. Furthermore, both methods may produce false predictions due to experimental deficiencies: for IHC-based classification, false negatives may arise from experimental or subjective errors detailed above. In fact it is possible that the observed discordance between ER status calls in primary and recurrent breast cancer is in some cases due to errors in IHC based classification rather than a reflection of a true change in tumour biology[12]. Direct comparison of the two methods reveals discrepancies but does not establish which method is more accurate.

We have chosen to investigate whether ER status defined by gene expression-based approaches or IHC produces more homogeneous patient cohorts in terms of clinical behaviour and outcome. We focus on clinical outcome in patients treated with and without endocrine therapy with discrepant ER classification in order to estimate and define the potential impact of false negative ER classification status on prognosis.

## Materials and Methods

This study was conducted in compliance to Dana Farber/Harvard Cancer Center, Institutional Review Board, protocol 93-085 with appropriated patient consent.

### Expression data sets and clinical annotation

For derivation of the genes specifically expressed in epithelium, we performed gene-expression profile analysis in breast cancer cell lines and sorted tumour epithelial cells. The data is publicly available from “Gene Expression Omnibus” (GEO) with accession number GSE23640 (http://www.ncbi.nlm.nih.gov/geo/query/acc.cgi?acc=GSE23640).

For meta-analyses, we chose ten data sets with pathological annotation of ER status based on IHC, EIA or both, incorporating a total of 1975 breast cancer specimens to derive and validate the classifiers for ER status (Table 1). These data sets were from independent studies and all publicly available[8], [10], [13]–[20]. Redundant samples were excluded according to the annotated sample origins.

**Table 1:** Summary of breast cancer cohorts used in this study.

	Number of patients	ER positive tumors (%)	Experiments for detecting ER expression	Received hormone/adjuvant therapy (%)	Follow-up timeMedian (range) in years
DFCI	175	100 (57.14)	IHC	NA	NA
MSK	99	57 (57.58)	IHC	NA	NA
EMC	286	209 (73.08)	10%, IHC;10 fmol/mg, EIA	0 (0)	7.17 (0.17–14.25)
JBI1	189	149 (78.84)	IHC	64 (33.86)	6.02 (0.02–14.53)
JBI2	120	0 (0)	IHC	120 (100.00)	NA
GIS	289	211 (73.01)	0.05 fmol/µg, EIA	147 (50.87)	9.92 (0–12.75)
JBI3	198	134 (67.68)	IHC	198 (100.00)	12.50 (0.40–24.95)
MDA1	133	82 (61.65)	10%, IHC	133 (100.00)	NA
MDA/MAQC	100	61 (61.00)	10%, IHC	100 (100.00)	NA
NKI	295	226 (76.61)	10%, IHC	130 (44.07)	7.22 (0.05–18.34)
TAM	155	155 (100%)	IHC	155 (100.00)	5.32 (0.12–13.76)
VDX	136	136 (100%)	IHC	136 (100.00)	6.98 (0.63–15.83)

We used the DFCI data sets as the reference data for derivation of both expression-based ER classifiers. For the meta-analysis and Kaplan-Meier survival curves, we used selected samples from six validation sets (EMC, JBI1, GIS, NKI, TAM and VDX) [8], [15], [16], [18], [21], [22], of which both the treatment and follow-up data were available from the original studies. The survival analyses were performed separately for patients received systematic treatment and patients did not.

### ER classification based on mixed Gaussian model

We used the Affymetrix HGU133a/HGU133plus2 probe set “205225_at” to represent the expression level of ESR1. We first verified the bimodal distribution using coefficient of bimodality with a threshold of 0.555. Then we decomposed the bimodal distribution into two Gaussian distributions, which correspond to two specific ER expression statuses. Based on the two inferred distributions we derived a cohort-specific cut-off value for ESR1 using Mahalanobis distance which minimizes the estimated false positive rate (FPR) and the false negative rate (FNR).

### ER classification based on gene expression signature

In order to derive a multi-gene expression signature for ER status, we collected nine cell lines (mostly ER negative) and five primary tumours samples enriched for epithelial cells by digesting tissue and sorting cells using BerEp4 antibody coated beads and profiled mRNA expression using microarray[23]. Next we selected 17256 genes which were classified as “present calls” in more than 50% of the samples by “dChip” [24]. In order to exclude possible confounding effects, we removed a further 1142 genes which are highly expressed in stromal tissue from laser-capture microdissected data sets using mix-effects linear model. From the remaining genes we selected 258 genes of which the coefficient of bimodality was larger than 0.555. Finally we selected 23 genes of which the estimated false positive and false negative rates of discrimination were both below 0.05 in the DFCI data set. We assigned weights for these 23 genes by the signs of correlation coefficients between each gene and the IHC based ER status, +1 or −1, and defined this as an ER expression signature. When predicting a given microarray data set, we took the expression profile of the 23 genes, determined their cut-off values based on the bimodal distribution in the data set and compared the weighted expression level of the 23 genes to the cut-off values. If 12 or more genes exceed the pre-defined cut-off values the sample was then classified as ER positive and ER negative if 11 or fewer genes exceeded the pre-defined cut-off values.

### Survival analysis

We first performed meta-analyses in the subsets of the four reference cohorts (EMC, JBI1, GIS, NKI) which received no chemotherapy or endocrine therapy for IHC-based and expression-based ER statuses. Then with same method we assessed the prognostic power of MKI67 in five-year disease-free survival within ER positive and negative tumours in the four untreated reference cohorts based on the IHC-based and expression-based ER classifiers. In order to assess the clinical relevance of misclassified ER positive or ER negative samples, we combined the ER calls in the four reference cohorts and estimated the hazard-ratios of expression-based ER status within IHC-based ER positive and ER negative subsets, respectively. In order to assess prognostic relevance in tamoxifen treated cohorts, we combined three reference cohorts (JBI1, TAM and VDX) in which patients were classified as IHC ER positive and all received endocrine therapy (Tamoxifen). We estimated the hazard-ratio for ESR1-based and signature-based ER status, respectively.

## Results

### An ER classifier based on ESR1 expression level

The expression profiles of many of the known breast cancer gene markers such as ESR1, ERBB2 and AR have been shown to follow a strong bimodal distribution, which corresponds to different tumour subtypes[11], [25], [26]. Previous studies have demonstrated that bimodal distribution of gene expression can be used to stratify breast cancers into subtypes of distinct prognoses and associated to known pathological risk-covariates [27], [28]. Two important issues remain to be addressed systematically: firstly, how can bimodality and a corresponding optimal threshold be quantitatively defined, to select gene markers from expression profiles enabling patient stratification; secondly, how can the false negative rate be defined and minimised using these techniques and what is its resultant impact upon clinical outcome?

To resolve these questions, we have examined the distribution of the ESR1 gene in ten datasets (Table 1). In nine datasets containing both IHC-based ER positive and ER negative tumours, we observed a bimodal distribution of ESR1 expression, with coefficients of bimodality ranging from 0.619 to 0.776, whereas in the data set with only IHC ER negative tumours (JBI2) there was no visible bimodal distribution of ESR1 (coefficient of bimodality  = 0.412, Supplement Figure S1).

For each of the nine data sets, we used mixed Gaussian models to decompose the density of ESR1 expression into two normal distributions and then derived a dataset-specific, optimal threshold value by Mahalanobis distance-based discrimination (Supplement Figure S2 showing the distribution of ESR1 levels for the DFCI cohort, other data not shown). We compared the predicted ER status based on thresholding for ESR1 expression value to the annotated ER status based on IHC for the ten data sets and found that the two methods generated significantly concordant classification of ER status (90.7%, Fisher exact test *P*<2.20E-16, Table 2).

**Table 2:** Comparison of ER status determined by microarray-based classifiers to the ER status based on conventional IHC method in 1846 samples from 10 published data sets.

Expression-based ER status	ESR1 based ER+	ESR1 based ER-	P value	23-gene signature based ER+	23-gene signature based ER-	P value
IHC ER+	93.0%	7.0%	<2.20E-16	90.9%	9.1%	<2.20E-16
IHC ER-	15.1%	84.9%	<2.20E-16	21.8%	78.2%	<2.20E-16
Overall concordance	90.7%			87.3%		
Basal	7.2%	92.8%	<2.20E-16	3.9%	96.1%	<2.20E-16
Luminal A	99.8%	0.2%	<2.20E-16	98.6%	1.4%	<2.20E-16
Luminal B	99.4%	0.6%	<2.20E-16	96.3%	3.7%	<2.20E-16
Her2	36.3%	63.7%	<2.20E-16	48.7%	51.3%	<2.20E-16

### An ER classifier based on epithelial-specific genes with a bimodal distribution

Relying exclusively on the expression of a single gene (ESR1) or genes which are co-regulated with ESR1 may have at least three limitations. Firstly, the measurement of any single gene transcript can be corrupted by experimental artefacts such as cross hybridization of microarray probes[29]. Secondly, the detection of ESR1 transcripts does not necessarily indicate a fully functional ER protein or ER signalling pathway; and finally, epithelial cell ER expression status is transient rather than constant, therefore determining ER status by its expression in a relatively small subset of cells may introduce additional bias[30]. Instead, the identification of genes following a bimodal distribution, reflecting ESR1 expression in breast cancer cohorts, which are epithelial-compartment specific, may present a more robust strategy to develop an ER classifier.

Since ESR1 is only expressed in a proportion of epithelial cells, the identification of epithelial-specific genes is a prerequisite to define ER associated genes and a specific ER classifier. Therefore we first identified a list of genes that are expressed in a wide variety of breast cancer epithelial cell lines and tumour epithelial cells but not in cells usually associated with breast stroma, such as adipocytes, fibroblasts or lymphocytes. Since ESR1 alone may not be the only component which determines the status of the entire signalling pathway and may be subject to random effects, we further hypothesized that genes in an ER status dependent “signature” will show a similar tendency to bimodality. Therefore, we performed an exhaustive search of the entire epithelial gene list to identify a cohort of genes with independent bimodal distribution, of which ESR1 is also a member.

**Figure 1:**
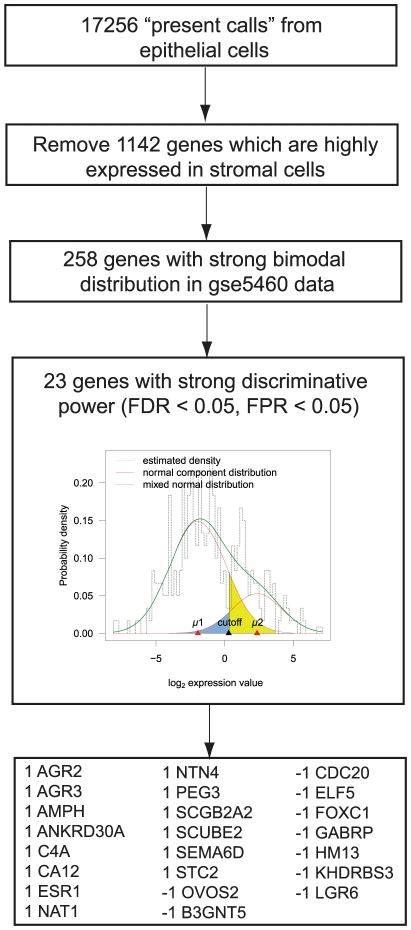
Schematic of deriving the 23-gene ER signature from epithelial specifically expressed genes.

We started with a set of 17256 genes with “present calls” in epithelial cell lines and enriched breast cancer epithelial cells. To improve the specificity of these genes, we eliminated 1142 genes which were found to be significantly over-expressed in tumour stromal cells relative to epithelial cells from microdissected biopsies[31]. Then we verified the bimodal distribution of these genes in the DFCI data set using standard deviation and coefficient of bimodality. Through this approach, we identified 258 genes for which the standard deviation across the samples is larger than 1 and the coefficient of bimodality is larger than 0.555[32]. These 258 genes included several epithelial markers such as KRT16, KRT23, KRT86 and MUC1 as well as several genes related to proliferation and the cell cycle, such as CDC20, CCNE2, CENPA, FOXA1 and FOXC1 (Figure 1).

To maximize discriminative power, we next estimated the false positive rate (FPR) and false negative rate (FNR) for each of the 258 genes based on their binary expression status determined by the bimodal distribution and the optimal threshold value, in the DFCI data set (Supplement Text S1). This filtering step yielded 23 genes with both FPR and FNR below 0.05. When combined into an ER status-associated gene expression signature, the weights of each gene were set to equal the sign of the correlation coefficient between the expression level of each individual gene and that of ESR1 expression. To validate the epithelial-specific gene selection approach and to confirm that the correlation of the 23 genes with ESR1 expression is unique to the epithelial gene list, we performed a similar procedure for bimodal expression of non-epithelial genes, the resulting gene list had a significantly lower correlation to IHC-based ER status in comparison to our 23 gene signature (Supplement Figure S3).

### Concordance between gene expression based ER classification and IHC based ER status

Using these approaches, we have developed two gene expression-based classifiers for ER status, the first was derived from the expression of ESR1 and the second was derived from a set of 23 genes specifically expressed within epithelial cells that were selected based on their bimodal distribution. We compared the ER status determined by these two classifiers to those based on IHC in the ten data sets. We found that both classifiers yielded highly concordant classifications with IHC-based ER status (Table 2). The classifier based on ESR1 expression level was concordant with IHC based ER status in 90.7% of the cases, and the classifier based on the 23-gene ER signature in 87.3% of the cases. On the other hand, both expression-based ER classification methods produced a notable level of discrepancy with IHC-based ER status; 9.3% and 12.7% of the samples are classified discordantly by ESR1 and 23-gene expression-based classifiers, respectively, compared to IHC-based ER status.

We also compared our two gene expression-based ER classifiers to the expression profiling-based classes of Perou et al[7]. As expected, tumours classified as ER negative by our gene expression classification methods showed overlap with the basal-like subtype of breast cancer (92.8%–96.1%). Moreover, expression-based ER positive tumours are significantly enriched for luminal A (98.6%–99.8%) and luminal B (96.3%–99.4%) subtypes (Table 2).

### Gene expression-based ER status classification as a prognostic tool

Since both IHC-based and expression-based ER classifiers may produce false predictions due to possible technical limitations, an evaluation of each classification method can be performed based on their association with clinical outcome. We performed a meta-analysis for ER status determined by IHC and by expression-based classifiers in four publicly available data sets including patients with both ER positive and ER negative breast cancer who received no hormone or adjuvant chemotherapy. Since the hazard ratio associated with ER status is time-dependent, we confined our analysis to a maximum follow up time of 2 years in which ER negative status is associated with a significantly higher clinical risk than ER positive status, especially when adjuvant chemotherapy is not implemented[33].

**Figure 2:**
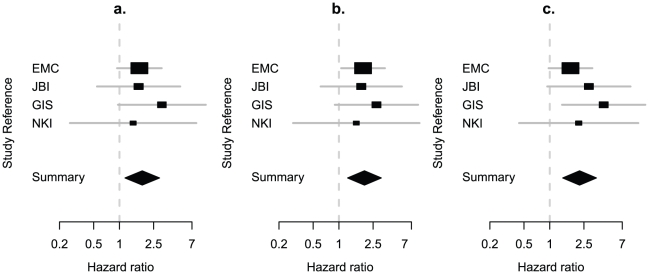
Prognostic power of IHC-based and expression-based ER status. Meta-analysis in four cohorts suggests that ER-statuses based on expression profile indicates stronger prognostic power than IHC based ER-status. The hazard ratios were estimated based on ER status determined by IHC (a.), ESR1 expression (b.) and 23-gene signature (c.), separately.

ER negative tumours are associated with worse clinical outcome compared to ER positive disease. Accurate estimate of the hazard ratio between ER negative tumours and ER positive tumours remains difficult to estimate due to the potential for misclassification by different classifiers. However, if the discordance in expression-based ER classification is due to real biological differences, the corresponding hazard ratio should be statistically significant compared to hazard ratios estimated from random flipping of original IHC ER calls. Our meta-analysis based on bootstrapping four reference data sets suggested that this is indeed the case. Both gene-expression-based ER classifications yielded higher hazard ratios in ER negative compared to ER positive tumours than IHC based ER status when assessing risk of disease recurrence (disease-free survival) (IHC based ER status HR = 1.84, 95% CI: 1.16–2.93, Figure 2a; ESR1 based: HR = 1.98, 95% CI: 1.26–3.12, Figure 2b; 23-gene ER signature based: HR = 2.31, 95% CI: 1.47–3.63, Figure 2c). The improved prognostic power by the 23-gene signature-based ER classification was significant based on the distribution of hazard ratios resulting from random re-sampling of the reference data sets (P = 0.006), but the one by ESR1 expression-based ER status was not (P = 0.053).

Another important clinical feature specific to ER status is the prognostic power associated with markers of proliferation which prognosticate in ER positive tumours but not in ER negative tumours [10]. Therefore an ideal ER classification system should maximize the difference between hazard-ratios for proliferation between ER positive and ER negative tumours. To assess this we performed a meta-analysis of the four reference cohorts for the prognostic power of proliferation in five-year disease-free survival. We estimated the hazard ratios of MKI67 expression, as a surrogate index of proliferation, in ER positive and ER negative subsets of the cohorts separately, based on both IHC-based and expression based ER classification. The hazard ratios of MKI67 expression are consistently higher in both ESR1 expression-based (HR = 3.45, 95% CI: 2.08–5.73) and 23-gene signature based ER positive tumours) (HR = 3.99, 95% CI: 2.31–6.89) compared to IHC-based ER positive tumours (HR = 2.33, 95% CI: 1.48–3.68) (Supplement Table S1); Bootstrapping results suggested that the improved prognostic power of MKI67 was significant for both ESR1-expression defined ER positive tumours (P<0.001) and for the 23-gene expression signature defined ER positive tumours (P<0.001). In ER negative tumours however, regardless of the classifiers applied, MKI67 manifested no significant prognostic power. To verify that the ER status-dependent prognostic power of proliferation is not affected by bias in tumour stage, we combined the ER calls in the four reference cohorts and performed a multivariate Cox regression based on lymph-node status, ER status, MKI67 expression and their interaction. The results were highly consistent with our previous analyses; ER negative status and MKI67 expression each holds independent, significant prognostic power in five-year disease-free survival, as well as the interaction between the two, which corresponds to the dependence of the prognostic power of MKI67 on ER receptor status. Moreover, the estimated hazard ratios of ER negative status, MKI67 expression and their interaction are consistently higher when expression-based ER status is used compared to IHC-based ER status (Table 3).

**Table 3:** Multivariate Cox regression of lymph node status, ER status, proliferation (MKI67) and their interaction in four reference cohorts with regard to IHC-based, ESR1-expression based and 23-gene signature based ER status.

ER classification	IHC-based ER(N = 995)	ESR1 expression based (N = 1004)	23-gene signature based (N = 1004)
LN positive	0.98 (0.73–1.31)P = 0.87	0.97 (0.72–1.29)P = 0.82	0.96 (0.72–1.29)P = 0.79
ER negative	1.67 (1.25–2.24)P = 0.00059	1.78 (1.36–2.35)P = 5.11E-5	1.98 (1.51–2.61)P = 9.05E-7
MKI67 expression	2.76 (1.76–4.06)P = 2.82E-7	3.35 (2.15–5.23)P = 9.80E-8	3.58 (2.25–5.70)P = 7.94E-8
ER-:MKI67 interaction	0.32 (0.16–0.63)P = 0.0011	0.24 (0.12–0.47)P = 2.80E-5	0.20 (0.10–0.39)P = 3.47E-6

LN: lymph node status.

### Minimising False Negative ER classification using complementary ER status expression-based classifiers

**Figure 3:**
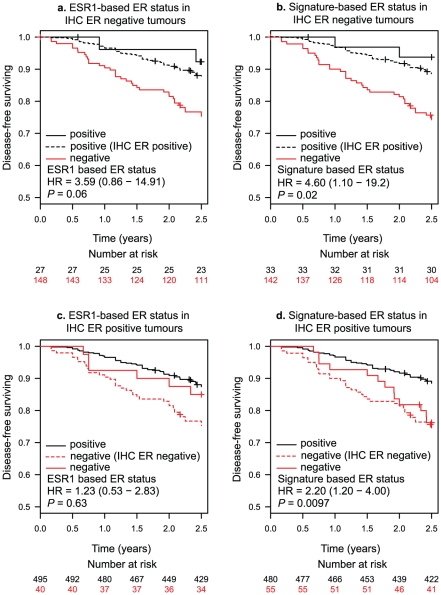
Expression-based ER classifiers reveal misclassification of IHC-based ER status which associated with clinical outcome. KM curves of disease-free survival showing IHC based ER- tumours separated by (a.) ESR1 expression-based and (b) 23-gene signature-based ER status; false ER- (23-gene signature based ER+, IHC based ER-) showed significantly better outcome which is close to true ER+ (23-gene signature based ER+, IHC based ER+, black dashed-line). And IHC based ER- tumours are separated by (c.) ESR1 expression and (d.) 23-gene signature based ER status; false ER+ (23-gene based ER-, IHC based ER+) showed significantly worse outcome which is close to true ER- (23-gene signature based ER-, IHC based ER-, red dashed-line).

Data presented here indicate a discrepancy between expression-based and IHC-based ER classifiers that affects between 9.3% and 12.7% of patients (Table 2). In order to assess the clinical impact of this potential false negative IHC discrepancy on clinical outcome, we compared disease-free survival in this subset of patients defined by expression-based or IHC-based ER status. Patients stratified with IHC-based ER negative disease but with 23 gene ER signature-based ER positive disease, had a significantly better outcome than those that were ER negative by both methods (Figure 3a, b). Next we assessed the outcome of patients classified as ER positive by IHC but as ER negative by expression-based classifiers. Patients stratified with IHC-based ER positive disease but 23 gene ER signature-based ER negative disease had a significantly worse outcome than their counterparts with matching IHC and 23-gene signature based ER positive status (Figure 3c, d). These results, together with the previous survival analyses, support the occurrence of false positive and negative ER status classification by IHC, which may be more efficiently classified with respect to prognosis using the 23-gene ER expression signature.

### Gene expression based ER status classification is associated with breast cancer outcome in patients that received tamoxifen treatment

An important clinical implication of ER status is the benefit patients derive from endocrine therapies such as tamoxifen. Given the discrepancy between IHC-based ER status and expression-based ER status, we reasoned that some of the variation seen in the clinical benefit from tamoxifen treatment may be partially attributable to true ER negative tumours being misclassified as ER positive. To test this, we collected three cohorts of IHC-defined ER positive patients with a total of 458 patients who had also received tamoxifen. We examined the ability of our expression-based classifiers to define outcome in these cohorts by meta-analysis and KM curves.

**Figure 4:**
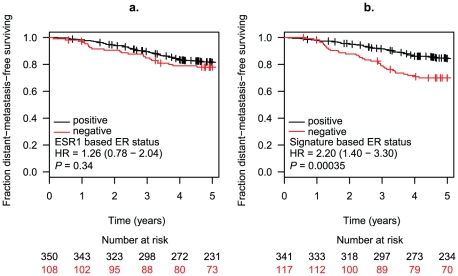
Expression-based ER classifiers predict distant-metastases-free survival of IHC ER positive patients treated with tamoxifen. (a.) ESR1 expression based ER status; (b.) 23-gene signature based ER status.

In the meta-analysis of IHC-defined ER positive patients, whilst ESR1 based ER classification was not associated with significant higher risk of relapse (HR = 1.39, 95% CI: 0.86–2.23), 23-gene signature-based ER negative tumours within this cohort were correlated with a poorer distant-metastasis-free survival over 5 years relative to the ER positive tumours defined by both IHC and the 23-gene signature (HR = 1.98, 95% CI: 1.19–3.28) (Supplement figure S4). KM curves combining all three cohorts suggested the same tendency, where 23-gene signature-based ER negative but IHC based ER positive tumours were associated with a poorer clinical outcome compared to ER positive tumours predicted by both methods (HR = 2.20, 95% CI: 1.40–3.30, P = 0.00035) (Figure 4b). Therefore ER status defined by the 23-gene signature may identify IHC ER positive tumours at higher risk of relapse following tamoxifen therapy. Taken together, these data suggest that expression-based ER status classifiers identifies clinically relevant associations with patient outcome both with and without endocrine therapy in those patients with discrepancy between immunohistochemistry and gene expression-based ER classification methods.

## Discussion

Improving the molecular classification of tumours with respect to predictive or prognostic biomarkers is essential for appropriate stratified and personalised medical approaches. False ER positive or false ER negative calls will result in futile or insufficient therapy for patients subject to tumour misclassfication. Given the substantial benefit of adjuvant endocrine therapies in ER positive disease, strategies to minimise the false negative ER status call rate are of paramount importance to prevent patients being denied such effective therapy[34]. Therefore, it is important to consider additional classification methods that might contribute to improving the reliability of ER testing in parallel with immunohistochemistry. Here we have presented one such approach that relies on the expression of a group of rationally selected, ER status associated genes. The implications of this work are that in these historical retrospective cohorts between 15.1% and 21.8% patients of IHC-based negative ER status would be classified with ER positive breast cancer using expression-based methods. It is likely that with improvements in contemporary ER assessment protocols and standardised immunohistochemistry procedures that this represents a significant over-estimation of the true false negative rate.

We are not suggesting that our method should replace traditional ER classification techniques, nor that this expression-based should be considered for re-stratifying IHC-defined ER positive disease as ER negative. Instead we suggest that expression-based methods should be considered for prospective assessment as a strategy, complementary to IHC, to minimise the potential for false negative ER status classification. Prospective analyses might investigate the potential benefit of tamoxifen in patients with ER IHC negative breast cancer whose disease is classified as ER positive by the 23-gene ER signature.

One of the major advantages of IHC-based ER status determination is that false negative ER calls, due to stromal contamination and the lack of cancer cells in the material examined, can be minimized[35], [36], [37]. In an attempt to minimise the risk of false negative ER calls from our gene signature methods due to stromal contamination, we have taken advantage of the fact that in ER negative tumours, not only is the set of ER regulated genes down-regulated but a well defined set of epithelial genes, such as FOXC1 and GABRP are overexpressed as well.

Therefore, the combined set of genes that correlate and anticorrelate with ER status may provide a reliable way of determining ER status in tumour cells whilst minimizing the risk of false negative calls due to excessive stromal contamination. In order to further reduce the risk of false ER calls by the 23-gene test, metagenes specifically measuring different tissue types could be used to correct for bias caused by other contaminations in the tumour samples[28].

The utility of PCR based multigenic outcome predictors has been demonstrated previously[38]. When considering practical realization it is important to note that the number of genes used in our ER status classifier is comparable to that used, for example, in the case of Oncotype recurrence score[38]. Therefore, if prospective studies of this multigene predictor in combination with IHC determine clinical utility that is complementary with IHC-based classification methods, it is reasonable to expect that this gene expression based method, especially in the case of questionable cases, may be added to the histopathological and molecular classification of breast cancer. We are currently planning to prospectively evaluate the benefit of such a test to minimise immunohistochemistry-defined false negative oestrogen receptor status classification.

### Conclusions

We have demonstrated that ER expression status determined from microarray data enables more accurate determination of clinical outcome of breast cancer in multiple reference cohorts. Our methods provided a set of gene markers which stratify breast cancer in terms of hormone receptor expression status and may further help to understand the biological background of heterogeneity of breast cancer. Moreover, with this 23-gene signature based ER classification method we have distinguished a subset of IHC ER positive breast cancer patients that have a poorer outcome following endocrine therapy that may be attributable to false positive classification of ER status by current histopathological methods. With future efforts, our approach may provide a new multi-gene assay to improve clinical stratification of hormone receptor positive breast cancer.

## Supporting Information

Figure S1 **Verification of bimodal distribution of ESR1 mRNA expression.** a. DFCI; b. MSK; c. MDA1; d. MDA/MAQC; e. UCSF; f. NKI; g. EMC; h. IJB1 and i. GIS.
(EPS)

Figure S2 **Bimodal distribution of ESR1 is associated with IHC ER status.** ESR1 (“205225_at”) expression levels follow a bimodal distribution in DFCI data set (left) and the inferred cutoff value based on the bimodal distribution is highly consistent with ER status determined by IHC (right).
(EPS)

Figure S3 **Epithelial genes with bimodal distribution indicated higher correlation to IHC-based ER status.**
(EPS)

Figure S4 **23-gene signature based ER status is associated with clinical outcome of tamoxifen-treated breast cancers.** Meta-analysis in three cohorts suggests that ER-statuses based on 23-gene expression signature indicate stronger predictive power than IHC based ER-status. The hazard ratios were estimated based on ER status determined by (a.) ESR1 expression and (b.) 23-gene ER signature, separately.
(EPS)

Table S1 **Meta-analyses of four reference cohorts for the prognostic power of proliferation (MKI67) within ER positive and negative tumours as classified by IHC-based, ESR1-expression based and 23-gene signature based classifiers.**
(DOC)

Text S1 **Supplementary method**.
(DOC)
