# Supplementary material for: Minimising Immunohistochemical False Negative ER Classification Using a Complementary 23 Gene Expression Signature of ER Status
Source: PLoS One. 2010 Dec 1;5(12):e15031. doi: 10.1371/journal.pone.0015031 (PMC2995741; doi:10.1371/journal.pone.0015031)
Supplement: Table S1 — Meta-analyses of four reference cohorts for the prognostic power of proliferation (MKI67) within ER positive and negative tumours as classified by IHC-based, ESR1-expression based and 23-gene signature based classifiers. (DOC) [file pone.0015031.s005.doc]

Table S1. Meta-analyses of four reference cohorts for the prognostic power of proliferation (MKI67) within ER positive and negative tumours as classified by IHC-based, ESR1-expression based and 23-gene signature based classifiers.

| ER classification | IHC based | ESR1 expression based | 23-gene signature based |
| --- | --- | --- | --- |
| ER+ | 2.33(1.48 – 3.68) | 3.45 (2.08 – 5.73) | 3.99 (2.31 – 6.89) |
| ER- | 0.87 (0.46 – 1.64) | 0.82 (0.45 – 1.49) | 0.62 (0.34 – 1.14) |
