# Supplementary material for: Minimising Immunohistochemical False Negative ER Classification Using a Complementary 23 Gene Expression Signature of ER Status
Source: PLoS One. 2010 Dec 1;5(12):e15031. doi: 10.1371/journal.pone.0015031 (PMC2995741; doi:10.1371/journal.pone.0015031)
Supplement: Text S1 — Supplementary method. (DOC) [file pone.0015031.s006.doc]

**Supplement Method**

**ER classification based on mixed Gaussian model and Mahalanobis distance**

For a given Affymetrix data set, we retrieved a vector of ESR1 expression levels measured by the probe set “205225_at”. We used histogram to represent the distribution of ESR1 expression and decomposed it into two Gaussian distributions *g*_1_ and *g*_2_.

Here *f*(*x*) is the observed distribution of ESR1, which is given by the frequencies as computed above. *β*_1_ and *β*_2_ are the coefficients of the two component Gaussian distributions, which correspond to the positive and negative ER status. In this study we estimated the parameters of the two Gaussian distributions (mean *µ* and standard deviation *σ*) using expectation maximization (EM) algorithm (Supplement Fig. 1).

Given the estimated component distribution of *g*_1_~*N*(*µ*_1_, *σ*_1_^2^) and *g*_2_~*N*(*µ*_2_, *σ*_2_^2^), we determined the cut-off value as the point *x* from which the Mahalanobis distance *D*_M_(*x*) to *g*_1_ and *g*_2_ are equal. That is:

$$\begin{matrix} D_{1\text{M}}(x)=(x-\mu_{1})/\sigma_{1} \\ D_{2\text{M}}(x)=(x-\mu_{2})/\sigma_{2} \\ D_{1\text{M}}=D_{2\text{M}} \\ \mu_{1}<x<\mu_{2} \end{matrix}$$

Solving the above equations we derived the cutoff value *c* for ESR1:

$$c=\frac{\sigma_{1}\mu_{2}+\sigma_{2}\mu{}_{1}}{\sigma_{1}+\sigma_{2}}$$

Finally, we estimated the probability of false positive predictions and false negative predictions that may arise from the chosen cut-off value *c*. The false positive rate (FPR) is given by the probability that a sample is from the *g*_1_ but the expression of ESR1 is higher than *c*, hence

$$\text{FPR}=\int_{c}^{+\infty} g_{1}(x)dx$$

Similarly, the false negative rate (FNR) of the classifier is the probability that a sample is from the *g*_2_ but the expression of ESR1 is lower than *c* (Supplement Fig. 1):

$$\text{FNR}=\int_{-\infty}^{c} g_{2}(x)dx$$

**ER classification based on gene expression signature**

Besides ESR1, we also expect other genes differentially expressed by epithelial cells are discriminative for ER status. We collected a set of cell lines (mostly ER negative) and a set of primary tumours samples enriched for epithelial cells by digesting tissue and sorting cells using BerEp4 antibody coated beads and profiled mRNA expression using microarray. All the samples were collected under Dana Farber/Harvard Cancer Center, Institutional Review Board, protocol 93-085 with patient consent. Next we selected 17256 genes which were classified as "present calls" in more than 50% of the samples by "dChip". In order to exclude possible confounding effects, we removed a further 1142 genes which are highly expressed in stromal tissue from laser-capture microdissected data sets using mix-effects linear model. For the remaining genes we computed the coefficient of bimodality *b* and standard deviation *σ* based on the expression profile of each gene *x*_i_ in the DFCI data set.

$$\begin{matrix} \sigma=\sqrt{\frac{1}{n}\sum_{i=1}^{n} (x_{i}-\overline{x})^{2}} \\ \text{Skewness }G_{1}=\frac{\sqrt{n(n-1)}}{n-2}\cdot\frac{\frac{1}{n}\sum_{i=1}^{n} (x_{i}-\overline{x})^{3}}{(\frac{1}{n}\sum_{i=1}^{n} (x_{i}-\overline{x})^{2})^{3/2}} \\ \text{Kurtosis G}_{2}=\frac{n(n+1)}{(n-1)(n-2)(n-3)}\cdot\frac{\frac{1}{n}\sum_{i=1}^{n} (x_{i}-\overline{x})^{4}}{\sigma^{4}}-3\frac{(n-1)^{2}}{(n-2)(n-3)} \\ b=\frac{G_{1}^{2}+1}{G_{2}+\frac{3(n-1)^{2}}{(n-2)(n-3)}} \end{matrix}$$

We selected 258 genes with *b* larger than 0.555 and *σ* larger than 1 and determined cut-off values for each gene based on mixed Gaussian distribution and Mahalanobis distance, as described above. From the 258 genes we further chose 23 genes of which the false positive rates and false negative rates of discrimination are both below 0.05 in the DFCI data set. We assigned weights for these genes by the signs of correlation coefficients between each gene and the IHC based ER status, +1 or -1, and defined as an ER expression signature. When predicting a given microarray data set, we took the expression profile of the 23 genes, determine their cut-off values respectively based on the bimodal distribution in the data set, then we compared the weighted expression level of the 23 genes to the cut-off values. If 12 or more genes exceed the pre-defined cut-off values the sample is then called ER positive, otherwise, ER negative.

**Survival analysis**

We first performed meta-analyses in the subsets of the four reference cohorts (EMC, JBI1, GIS, NKI) which received no chemo, radio or endocrine therapy for IHC-based and expression-based ER statuses. Next we merged the samples from the four untreated cohorts and plotted Kaplan-Meier survival curves for disease-free surviving based on the ER status from different classifiers, respectively. The hazard ratio of each classifier were estimated and compared to each other. In order to assess the clinical impact of misclassified ER status by IHC based method, we took IHC-based ER positive and ER negative subsets from previous combined cohort and estimated the hazard-ratios of ESR1 expression-based and the 23-gene signature based ER status, respectively.

As for predictive power, we combined three reference cohorts (JBI1, TAM and VDX) of which patients are classified as IHC ER positive and all received endocrine therapy (Tamoxifen). We estimated the hazard-ratio for ESR1-based and signature-based ER status, respectively.
